# Supplementary material for: Cis-Cardio: A comprehensive analysis platform for cardiovascular-relavant cis-regulation in human and mouse
Source: Mol Ther Nucleic Acids. 2023 Jul 27;33:655–67. doi: 10.1016/j.omtn.2023.07.030 (PMC10458290; doi:10.1016/j.omtn.2023.07.030)
Supplement: Document S1. Figures S1 and S2 and Table S2 [file mmc1.pdf]

OMTN, Volume *33*

Supplemental information

Cis-Cardio: A comprehensive analysis platform for cardiovascular-relavant *cis*-regulation in human and mouse

Chao Song, Yuexin Zhang, Hong Huang, Yuezhu Wang, Xilong Zhao, Guorui Zhang, Mingxue Yin, Chenchen Feng, Qiuyu Wang, Fengcui Qian, Desi Shang, Jian Zhang, Jiaqi Liu, Chunquan Li, and Huifang Tang

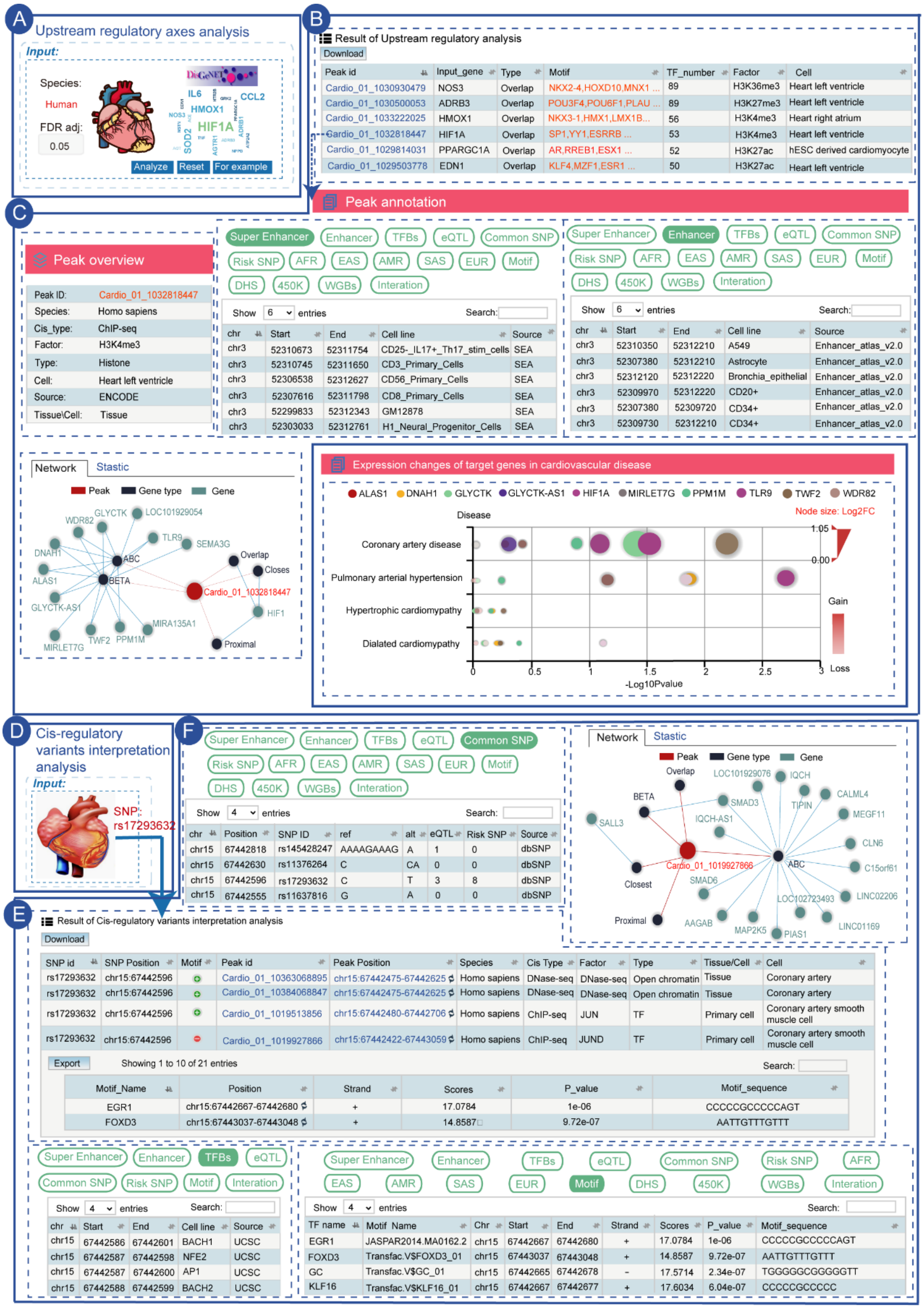

Figure S1: Additional case study related analysis results of Cis-Cardio. (A) Input genes of top 20 heart failure disease genes, which were downloaded from DisGeNET. (B) Results of Upstream regulatory axes analysis. (C) Data interface of ChIP-seq peak

of interest, including genomic locus, target gene assignment, target gene network, peak annotation and target gene annotation and differential expression information. (D) Input variant of coronary heart disease. (E) Results of Cis-regulatory variants interpretation analysis. (F) Data interface of ChIP-seq peak of interest, including TF binding sites, target gene assignment, target gene network and peak annotation.

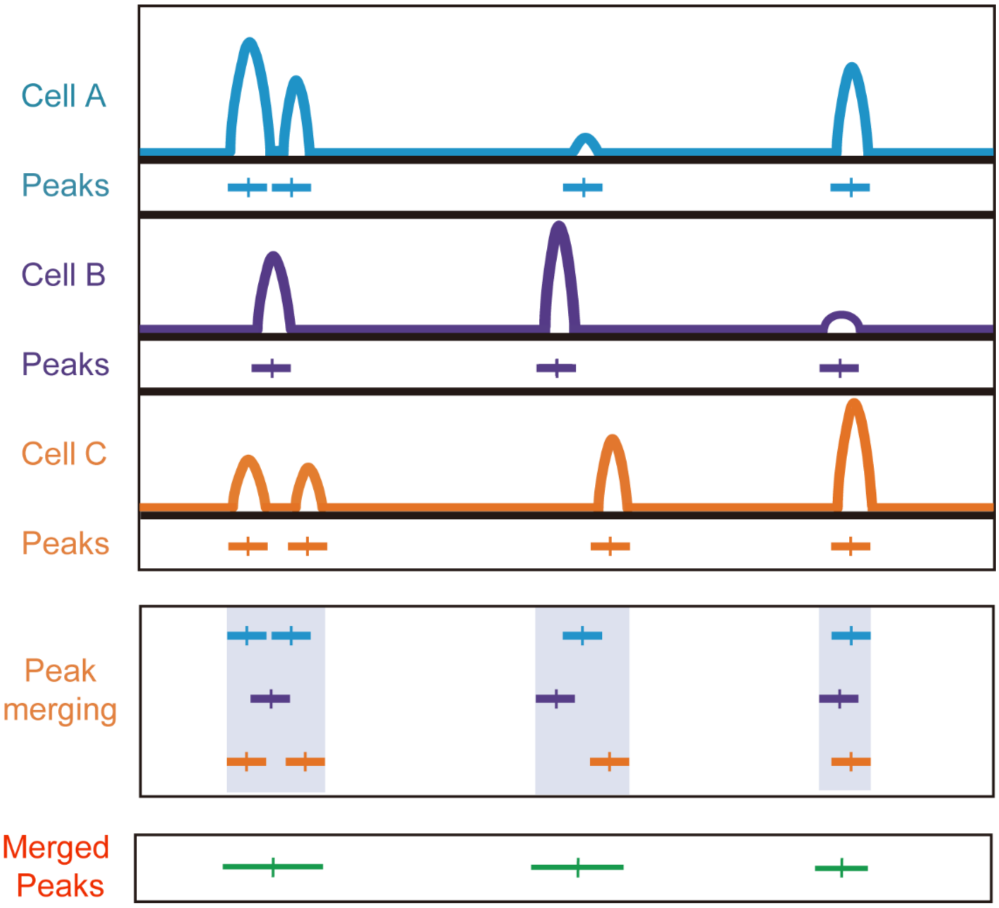

Figure S2: The protocol to merge peaks into an integrated region set based on Bedtools.

Table S1: Biosample metadata of Cis-Cardio. Sheet 1 is the metadata of samples with GEO accession number. Sheet 2 is the metadata of samples with ENCODE accession number.

Table S2: Metadata and processing protocol of (epi)genetics annotation data.

Type	Source name	Link	Version
Super Enhancer	SEdb	http://www.licpathway.net/sedb	V1
Super enhancer	SEA	http://sea.edbc.org	V3
Super enhancer	dbSuper	http://bioinfo.au.tsinghua.edu.cn/dbsuper/	V1
Enhancer	EnhancerAtlas	http://www.enhanceratlas.org/indexv2.php	V2
Enhancer	HACER	http://bioinfo.vanderbilt.edu/AE/HACER/	V1
Enhancer	FANTOM5	https://fantom.gsc.riken.jp/5/datafiles/latest/extra/Enhancers/	V1
Enhancer	DENDB	http://www.cbrc.kaust.edu.sa/dendb/	V1
Enhancer	ENdb	http://www.licpathway.net/ENdb	V1
Enhancer	ENCODE	https://www.encodeproject.org/search/?type=Experiment&status=released&award.rfa=ENCODE2	V2
Common SNP	dbSNP	http://www.ncbi.nlm.nih.gov/SNP/	dbSNP 150
risk SNP	GWASdb v2	http://jjwanglab.org/gwasdb	V2
eQTL	PancanQTL	http://bioinfo.life.hust.edu.cn/PancanQTL	V1
eQTL	seeQTL	http://www.bios.unc.edu/research/genomic_software/seeQTL/	V1
eQTL	SCAN	http://www.scandb.org	V1
eQTL	Oncobase	http://www.oncobase.biols.ac.cn	V1
Chromatin interaction	4DGenome	http://4dgenome.int-med.uiowa.edu/	V1
Chromatin interaction	Oncobase	http://www.oncobase.biols.ac.cn	V1
DHS	UCSC	https://www.genome.ucsc.edu/index.html	V1
DHS	ENCODE	https://www.encodeproject.org/search/?type=Experiment&status=released&award.rfa=ENCODE3	V3
DNA methylation	ENCODE	https://www.encodeproject.org/search/?type=Experiment&status=released&award.rfa=ENCODE3	V3
TFBS	UCSC	https://www.genome.ucsc.edu/index.html	V1

PPI	STRING	https://cn.string-db.org/	V11.5

Table S3: Pathway gene set of Cis-Cardio analysis panel.

Description of "bed format" download files

1) **Human bed file column name:** Chrome, Start, End, Peak_id,, Peak size, Number of super enhancer, Number of enhancer, Number of common SNP, Number of Risk SNP, Number of eQTL, Number of DHS, Number of 450k sites and Number of TFBS.
2) **Mouse bed file column name:** Chrome, Start, End, Peak_id,, Peak size, Number of super enhancer, Number of TFBS. Number of enhancer and Number of DHS.
