# Supplementary material for: Cis-Cardio: A comprehensive analysis platform for cardiovascular-relavant cis-regulation in human and mouse
Source: Mol Ther Nucleic Acids. 2023 Jul 27;33:655–67. doi: 10.1016/j.omtn.2023.07.030 (PMC10458290; doi:10.1016/j.omtn.2023.07.030)
Supplement: Document S2. Article plus supplemental information [file mmc4.pdf]

Molecular Therapy
Nucleic Acids
Original Article

Cis-Cardio: A comprehensive analysis platform for cardiovascular-relavant *cis*-regulation in human and mouse

Chao Song,[1,2,3,4,5,12] Yuexin Zhang,[1,2,3,4,5,12] Hong Huang,[1,2,3,5,9,12] Yuezhu Wang,[6] Xilong Zhao,[6] Guorui Zhang,[1,2,3] Mingxue Yin,[1,2,3] Chenchen Feng,[6] Qiuyu Wang,[1,2,3,4,5,7,8] Fengcui Qian,[1,2,3,4,5] Desi Shang,[1,2,3,4,5,7,8] Jian Zhang,[6] Jiaqi Liu,[1,2,3,4,5] Chunquan Li,[1,2,3,4,5,7,8,10,11] and Huifang Tang[1,2,3,4,5,9]

[1]The First Affiliated Hospital, Institute of Cardiovascular Disease, Hengyang Medical School, University of South China, Hengyang, Hunan 421001, China; [2]The First Affiliated Hospital, Cardiovascular Lab of Big Data and Imaging Artificial Intelligence, Hengyang Medical School, University of South China, Hengyang, Hunan 421001, China; [3]Hunan Provincial Key Laboratory of Multi-omics and Artificial Intelligence of Cardiovascular Diseases, University of South China, Hengyang, Hunan 421001, China; [4]School of Computer, University of South China, Hengyang, Hunan 421001, China; [5]The First Affiliated Hospital, Department of Cardiology, Hengyang Medical School, University of South China, Hengyang, China; [6]School of Medical Informatics, Daqing Campus, Harbin Medical University, Daqing 163319, China; [7]Department of Biochemistry and Molecular Biology, School of Basic Medical Sciences, Hengyang Medical School, University of South China, Hengyang, Hunan 421001, China; [8]Department of Cell Biology and Genetics, School of Basic Medical Sciences, Hengyang Medical School, University of South China, Hengyang, Hunan 421001, China; [9]Clinical Research Center for Myocardial Injury in Hunan Province, Hengyang, Hunan 421001, China; [10]National Health Commission Key Laboratory of Birth Defect Research and Prevention, Hunan Provincial Maternal and Child Health Care Hospital, Changsha, Hunan 410008, China; [11]Key Laboratory of Rare Pediatric Diseases, Ministry of Education, University of South China, Hengyang, Hunan 421001, China

Cis-regulatory elements are important molecular switches in controlling gene expression and are regarded as determinant hubs in the transcriptional regulatory network. Collection and processing of large-scale _cis_-regulatory data are urgent to decipher the potential mechanisms of cardiovascular diseases from a _cis_-regulatory element aspect. Here, we developed a novel web server, _Cis_-Cardio, which aims to document a large number of available cardiovascular-related _cis_-regulatory data and to provide analysis for unveiling the comprehensive mechanisms at a _cis_-regulation level. The current version of _Cis_-Cardio catalogs a total of 45,382,361 genomic regions from 1,013 human and mouse epigenetic datasets, including ATAC-seq, DNase-seq, Histone ChIP-seq, TF/TcoF ChIP-seq, RNA polymerase ChIP-seq, and Cohesin ChIP-seq. Importantly, _Cis_-Cardio provides six analysis tools, including region overlap analysis, element upstream/downstream analysis, transcription regulator enrichment analysis, variant interpretation, and protein-protein interaction-based co-regulatory analysis. Additionally, _Cis_-Cardio provides detailed and abundant (epi-) genetic annotations in _cis_-regulatory regions, such as super-enhancers, enhancers, transcription factor binding sites (TFBSs), methylation sites, common SNPs, risk SNPs, expression quantitative trait loci (eQTLs), motifs, DNase I hypersensitive sites (DHSs), and 3D chromatin interactions. In summary, _Cis_-Cardio is a valuable resource for elucidating and analyzing regulatory cues of cardiovascular-specific _cis_-regulatory elements. The platform is freely available at http://www.licpathway.net/Cis-Cardio/index.html.

INTRODUCTION

The cardiovascular system plays a vital role in transporting blood and nutrients around the body. Dysfunction or injury of the cardiovascular system can lead to poor prognosis of cardiovascular diseases, such as myocardial infarction, heart failure, and atherosclerosis. However, the molecular regulatory mechanism of these diseases is unclear, and it is urgent to unveil it to maintain normal physiological functions. Abnormal gene expression is a risk factor in the development of complex diseases and is essential for understanding the pathological mechanisms. Thus, our study aims to dissect the comprehensive gene regulation patterns in the cardiovascular system.

Gene expression programs are complex and are driven by transcription regulators that occupy at *cis*-regulatory elements, such as promoters and distal enhancers, thereby supervising the expression activity of downstream genes.[1–3] Mechanically, the mediator complex links signals from multiple regulators, such as transcription factor (TF) and transcription co-factor (TcoF), and recruits cohesin complexes to bind to RNA polymerase II and initiate gene transcription.[4,5]

Received 30 January 2023; accepted 25 July 2023;
https://doi.org/10.1016/j.omtn.2023.07.030.

[12]These authors contributed equally

Correspondence: Chunquan Li, The First Affiliated Hospital, Institute of Cardiovascular Disease, Hengyang Medical School, University of South China, Hengyang, Hunan 421001, China.
E-mail: lcqbio@163.com

Correspondence: Huifang Tang, The First Affiliated Hospital, Institute of Cardiovascular Disease, Hengyang Medical School, University of South China, Hengyang, Hunan 421001, China.
E-mail: tanghuifang999@163.com

This is an open access article under the CC BY-NC-ND license (http://creativecommons.org/licenses/by-nc-nd/4.0/).

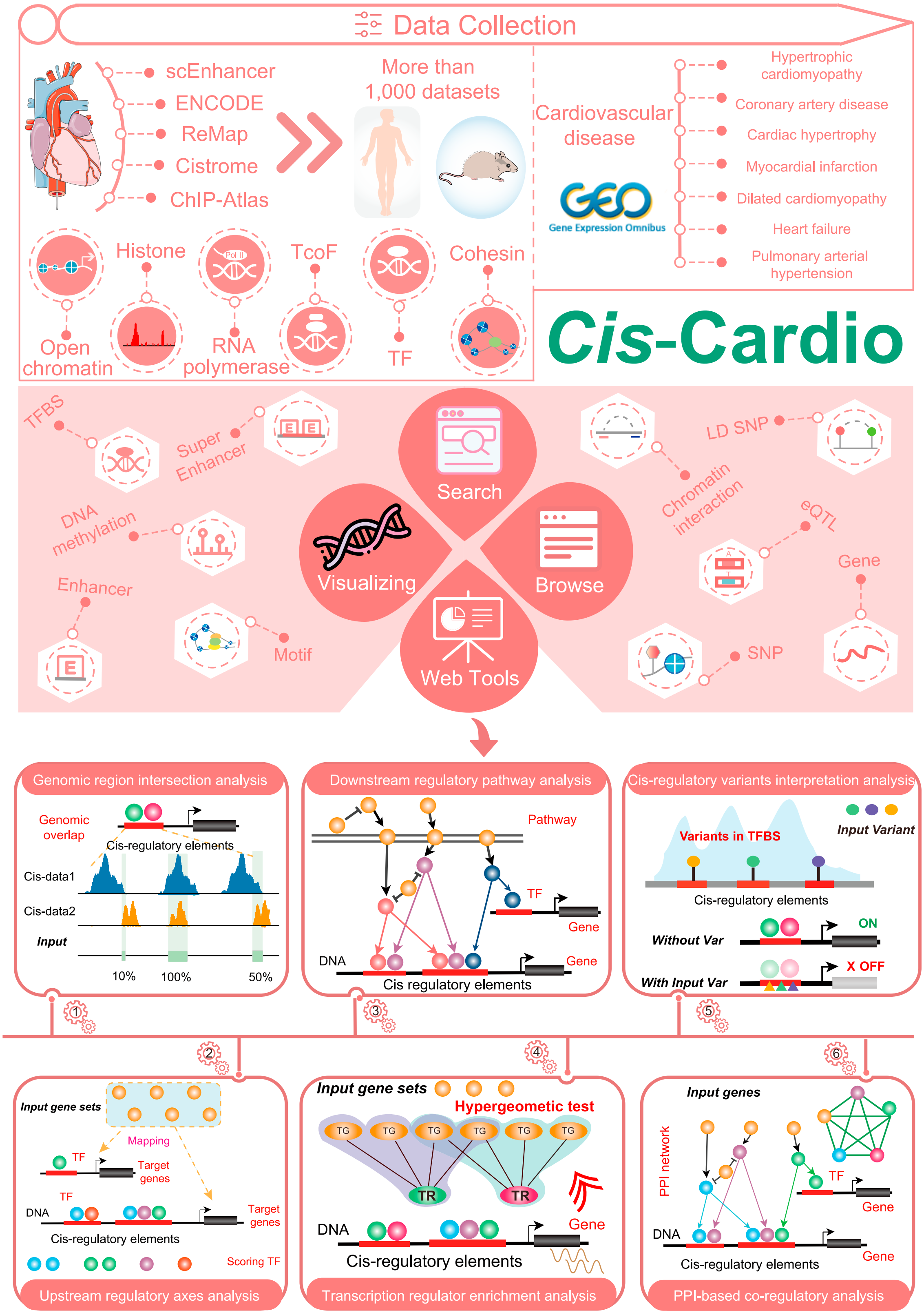

(legend on next page)

As the crucial commanders of gene expression, it is important to illustrate the downstream regulatory atlas of *cis*-regulatory regions.[6,7] Recently, Zheng et al. constructed the Cistrome Database that contains approximately 47,000 human and mouse samples from about 24,000 collected epigenetics datasets to dissect the global gene expression programs.[8] *cis*-regulation of gene expression also provides an important perspective to understand disease etiology.[9] In the field of cardiovascular diseases, some studies have also revealed core regulatory networks based on *cis*-regulation. For instance, Hocker et al. found >280,000 *cis*-regulatory regions of heart failure and annotated two variants that affect *cis*-regulatory regions controlling *KCNH2/HERG* expression and action potential repolarization in single-cell resolution.[10] Huang et al. found that super-enhancer-driven circRNA *Nfix* could promote cardiac regenerative repair by inhibiting *Ybx1* ubiquitin-dependent degradation and activating *miR-214* expression after myocardial infarction.[11] Galang et al. revealed an *Isl1* enhancer that regulates pacemaker cells development and sinoatrial node function via *cis*-regulation.[12] *Cis*-regulatory regions have the strong ability to recruit transcriptional regulators. Genome variations in TFs, cofactors, and chromatin regulator binding sites are also major causes of cardiovascular dysfunction, such as mutations in *GATA4*[13] and *TBX5*.[14] Therefore, identification and annotation of *cis*-regulatory regions are the central topics in transcription regulation. Moreover, investigating the downstream regulatory cues of *cis*-regulatory regions is also crucial to dissect the cardiovascular-specific gene expression pattern. These studies demonstrate the importance and widespread utility of *cis*-regulatory regions for addressing key regulatory cues associated with cardiovascular physiological and pathological processes.

Technologically, several high-throughput sequencing techniques, such as chromatin immunoprecipitation sequencing (ChIP-seq), assay for transposase-accessible chromatin sequencing (ATAC-seq), and DNaseI sequencing (DNase-seq) have been developed for identifying genome-wide *cis*-regulatory regions.[15] Based on these publicly available epigenomics datasets, some databases or web tools have also been developed to focus on understanding the regulatory potentials and biological functions of *cis*-regulatory regions, such as ENCODE, Cistrome, ReMap, ChIP-Atlas, SEdb2.0, and GREAT.[8,16–20] These resources have provided valuable data for *cis*-regulation studies. Moreover, single-cell transcription regulation data have also been released, such as single-cell ATAC-seq (scATAC-seq) from scEnhancer.[21] However, all these resources have paid more attention to provide genome-wide *cis*-regulatory regions and basic functions but have not focused on regulatory annotations, including comprehensive upstream and downstream regulatory annotations. Especially, the barrier to understanding the genetic and molecular basis of cardiovascular diseases is the paucity of resources to mark the cardiovascular-specific gene regulatory programs. Thus, it is highly desirable to construct an integrated resource and analysis tools of cardiovascular-related *cis*-regulatory regions, which provides comprehensive annotations of *cis*-regulatory regions and enables biologists to annotate, analyze, and understand these cardiovascular-related *cis*-regulatory regions.

To investigate the *cis*-regulatory mechanisms of the cardiovascular system, we developed the *Cis*-Cardio platform (http://www.licpathway.net/Cis-Cardio/index.html), which is a comprehensive server for analyzing human and mouse cardiovascular-related *cis*-regulatory elements. *Cis*-Cardio is designed to document and annotate a large number of cardiovascular-specific *cis*-regulatory elements and to uncover the comprehensive mechanisms in *cis*-regulation level. The current version of *Cis*-Cardio catalogs a total of 45,382,361 candidate *cis*-regulatory elements from over 1,013 human and mouse epigenetic datasets, including ATAC-seq, scATAC-seq, DNase-seq, Histone ChIP-seq, TF/TcoF ChIP-seq, RNA polymerase ChIP-seq, and Cohesin ChIP-seq. These datasets were manually curated from numerous epigenetic databases and almost covered all samples of cardiovascular systems, such as tissues, primary cells, and induced pluripotent stem cells. Emphatically, *Cis*-Cardio provides detailed and abundant (epi-) genetic annotations in *cis*-regulatory regions, such as super-enhancers, enhancers, transcription factor binding sites (TFBSs), methylation sites, common SNPs, risk SNPs, expression quantitative trait loci (eQTLs), motifs, DNase I hypersensitive sites (DHSs), and 3D chromatin interactions. *Cis*-Cardio also provides *cis*-element downstream target genes by mapping binding regions into genomes in three methods. Furthermore, *Cis*-Cardio provides various annotations for *cis*-element target genes, including pathways, Gene Ontology (GO) terms, and expression changes in major cardiovascular diseases. Especially, *Cis*-Cardio provides six types of *cis*-regulatory analyses for users, including genome region overlap analysis, upstream/downstream regulatory axes analysis, transcription regulator enrichment analysis, variant interpretation, and transcription co-regulatory analysis. *Cis*-Cardio is a user-friendly platform to analyze, query, browse, and visualize information associated with *cis*-regulatory elements. We believe that *Cis*-Cardio could become a useful and effective platform for exploring potential functions and *cis*-regulation in cardiovascular diseases.

RESULTS

Overview and characteristic of *Cis*-Cardio

The main framework and functions of *Cis*-Cardio are illustrated in Figure 1, including the collection of *cis*-regulatory regions from epigenetic datasets, (epi-) genetic annotations, disease gene expression, and the six analysis panels. Briefly, the current version of *Cis*-Cardio cataloged a total of 45,382,361 genomic regions from over 1,013 human and mouse epigenetic datasets (including ATAC-seq,

Figure 1. Data collection and construction of *Cis*-Cardio

The top area contains the data scope of the server, which includes collection of ATAC-seq, DNase-seq, Histone ChIP-seq, TF/TcoF ChIP-seq, RNA polymerase ChIP-seq, and Cohesin ChIP-seq. The middle area contains (epi-) genetic annotations in *cis*-regulatory regions. The bottom area contains six analysis tools for cardiovascular-related *cis*-regulation. *Cis*-Cardio is a user-friendly platform to analyze, query, browse, and visualize information associated with *cis*-regulatory elements.

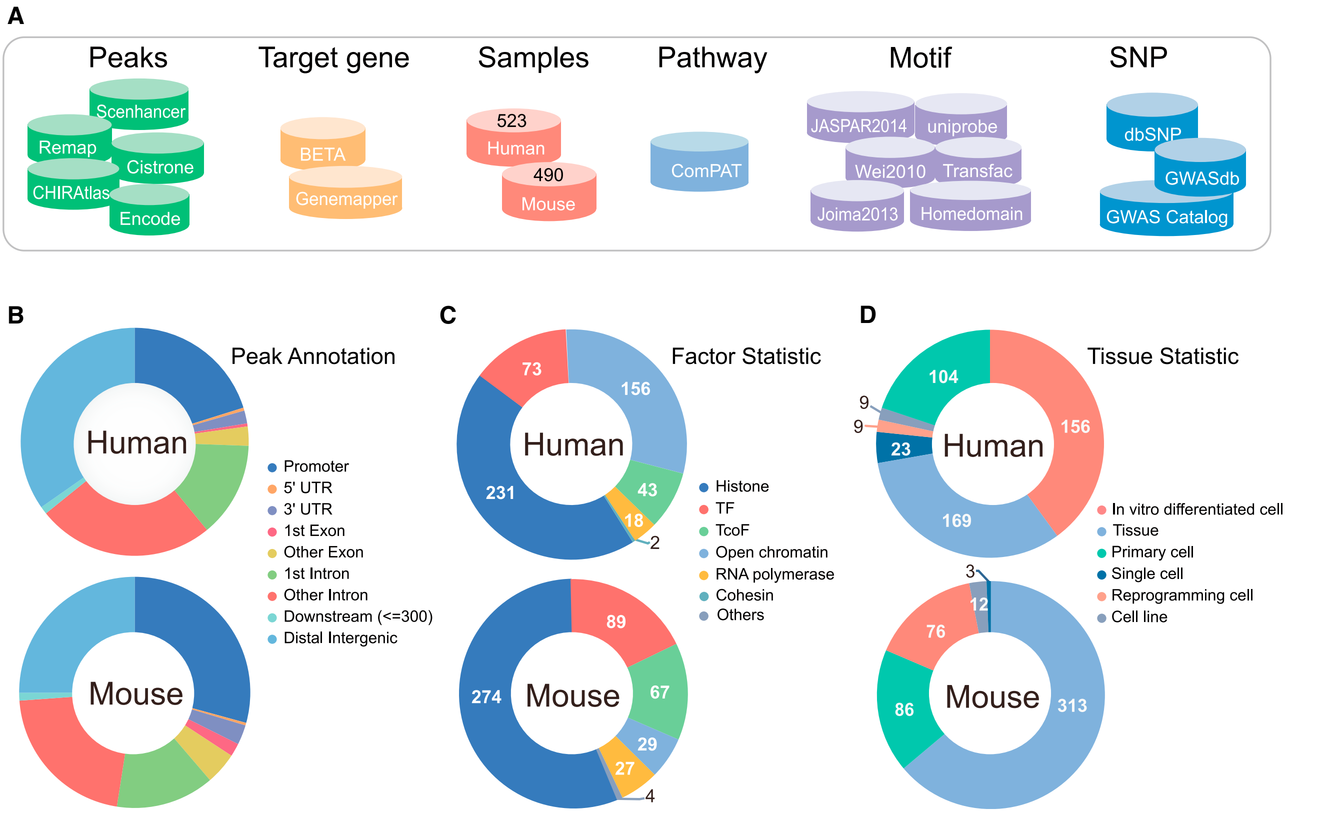

Figure 2. Statistics of contents of *Cis*-Cardio

(A) Data sources of *cis*-regulatory peaks, target genes, samples, pathways, motifs, and SNPs. (B) Global peak annotation distributions of human and mouse *cis*-regulatory peaks. (C) Global factor statistics of human and mouse samples. (D) Tissue type statistics of human and mouse samples.

scATAC-seq, DNase-seq, Histone ChIP-seq, TF/TcoF ChIP-seq, RNA polymerase ChIP-seq, and Cohesin ChIP-seq) of five resources. Modifications and variations of *cis*-regulatory elements play key roles in guiding gene expression. For example, super-enhancers or enhancers can recruit multiple transcription regulators, such as TFs and TcoFs, to form a transcription complex to participate in gene regulation.[22–24] Variations in these regulatory elements can disrupt the high binding affinity between transcription regulators and enhancers, leading to the dysfunction of downstream regulatory axes.[25] Chromatin interaction information between distal elements and proximal elements provides more evidence to identify the potential *cis*-regulatory elements. Additionally, DNA methylation level also determines the downstream gene expression. In the field of cardiovascular disease, previous studies have also demonstrated the significance of *cis*-regulatory patterns.[26] Therefore, for each *cis*-regulatory region, we integrated a large number of (epi-) genetic annotations and provided downstream gene annotations from multiple resources and strategies (Figure 2A).

Additionally, we performed peak annotation for all candidate *cis*-regulatory elements via ChIPseeker. Results showed that a majority of candidate *cis*-regulatory elements were located at the promoter and distal intergenic regions (Figure 2B). Hence, *Cis*-Cardio provided the detailed and abundant (epi-) genetic annotations of *cis*-regulatory regions, such as distal super-enhancers, distal enhancers, TFBSs, methylation sites, common SNPs, risk SNPs, eQTLs, motifs, DHSs, and 3D chromatin interactions. Downstream target genes, pathways, GO terms, and expression changes in major cardiovascular diseases were also provided. Importantly, *Cis*-Cardio provided six analysis tools to help users decipher the multi-omics regulatory networks of cardiovascular diseases. Moreover, *Cis*-Cardio covered almost transcription regulators and cell/tissue types in the field of cardiovascular disease (Figures 2C and 2D). Summarily, *Cis*-Cardio is a user-friendly web server to analyze, browse, and visualize information associated with *cis*-regulatory elements.

Case study of *Cis*-Cardio

Cis-Cardio provided six analysis tools to dissect the candidate *cis*-regulatory element-mediated transcription regulation mechanism of cardiovascular diseases. To illustrate the use and analysis performances of *Cis*-Cardio, we performed four case studies by integrating cardiac-specific genes to enrich upstream transcription regulators, inputting cardiac-specific super-enhancers to locate the super-enhancer-related regulatory axis and locating regulatory information for cardiovascular-related SNP sites.

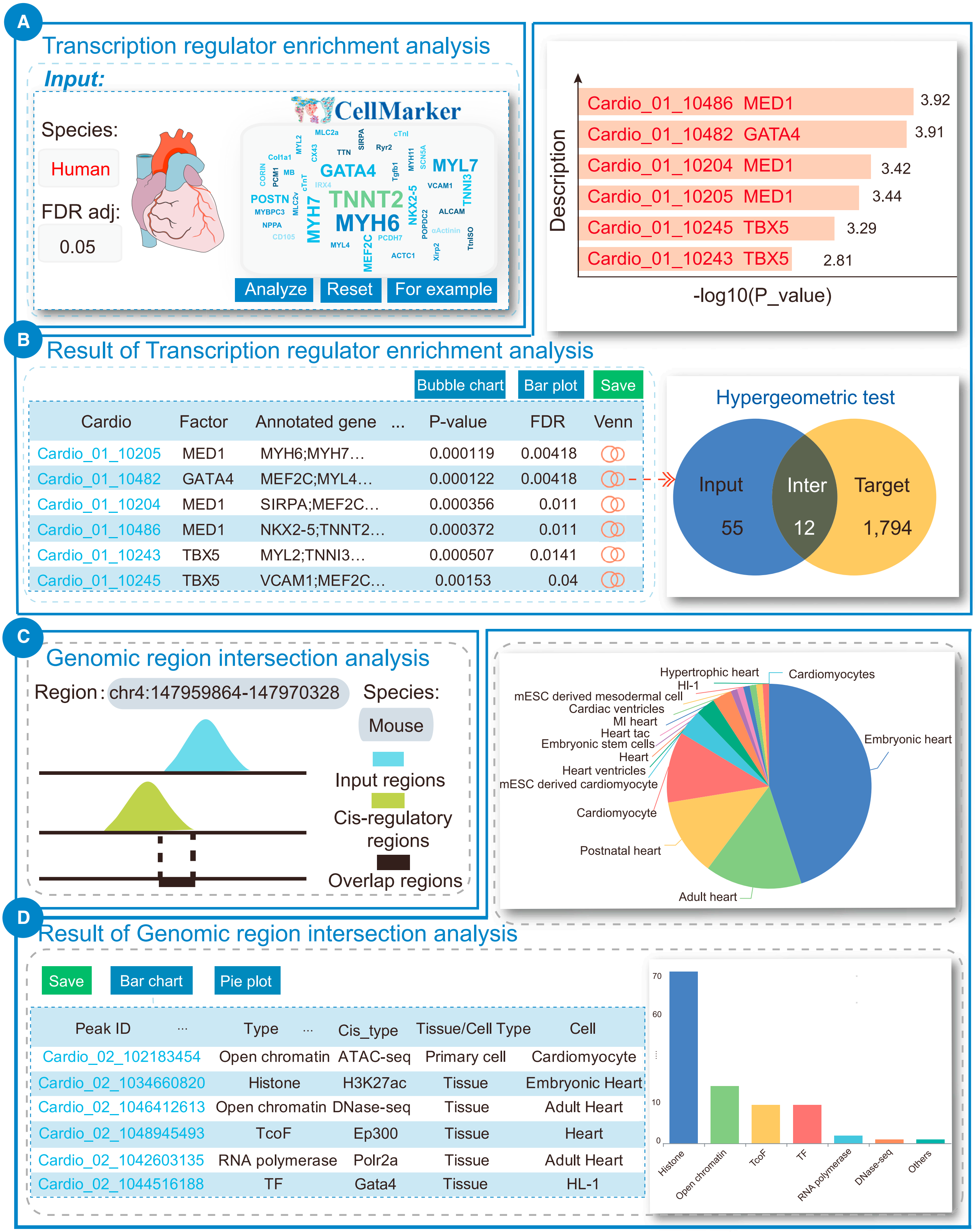

(legend on next page)

Transcription regulator enrichment analysis for marker gene set of cardiac tissue

The transcription regulator is the key supervisor of transcription regulation, which can regulate downstream gene expression by binding DNA regulatory elements and formatting chromatin loops. The activity of epigenetics factors also plays an important role in the regulatory processes. Notably, compared with gene expression or gene-set-based TF enrichment analysis, ChIP-seq-based methods have the advantages in prediction accuracy and tissue specificity. Here, we integrated all the regulatory factor-gene pairs of all epigenetics data and used a hypergeometric test to identify the potential upstream regulators. As an example, we first collected the 55 human cardiac-specific genes from CellMarker,[27] which included the typical markers *GATA4*, *NKX2-5*, *TNNT2*, *MYH6*, and *MYH7* (Figure 3A, left). Then we set the analysis parameters as default and clicked "Analyze" to perform enrichment analysis. On the results page, *Cis*-Cardio will list all the ranked regulators based on hypergeometric test. Detailed enrichment analysis statistics and annotated genes are also provided for users to optimize the transcription regulator-target gene relationships (Figure 3B).

As a result, some potential upstream regulators were identified with top-ranking statistical p value, such as *MED1*, *GATA4*, and *TBX5* (Figure 3A, right), which was in coincidence with previous studies. Ang et al. demonstrated that *GATA4* broadly co-occupied cardiac super-enhancers with *TBX5*.[13] Mutation with *GATA4* could decrease *TBX5* recruitment to cardiac super-enhancers, leading to the dysregulation of downstream genes and phenotypic abnormalities. *MED1* is a typical chromatin mark of super-enhancers.[28] These results suggested that *MED1-GATA4-TBX5* complex could occupy in super-enhancers to maintain cardiomyocyte identity. Moreover, results showed that *GATA4* overlapped more target genes than other regulators, including validated targets *NPPA* and *NPPB*, suggesting the capacity of *GATA4* in regulating cardiac marker genes. Above all, *Cis*-Cardio could identify key transcription regulators of a gene set, suggesting the usefulness in exploring the cardiovascular-specific transcription regulation mechanisms.

Identification of the overlapped candidate *cis*-regulatory elements for *Nppa*/*Nppb* super-enhancer

Cis-Cardio processed and curated a large number of *cis*-regulatory regions from diverse epigenetics data, which covered the vast majority of cardiovascular-specific active chromatins. We provided the genomic region intersection analysis to identify the overlapped region of interest from background epigenetics annotation data. Super-enhancers, which are marked by *H3K27ac*, *EP300*, *MED1*, and *BRD4*, are the representative regulatory elements in maintaining cell identity. Studies revealed that super-enhancers exert functions by recruiting numerous transcription regulators. Here, we collected and analyzed the mouse super-enhancer region of heart failure disease genes *Nppa/Nppb*[29] (Figure 3C, left). *Cis*-Cardio listed all the information of each overlapped region, including genomic locus information, epigenetics data type, and tissue/cell information (Figure 3D, left). Users can click the Peak_ID to view the detailed information of regions of interest.

As a result, 577 *cis*-regulatory regions were identified to overlap with the input super-enhancer, indicating the regulatory importance of the super-enhancer in cardiovascular diseases. Based on the cell fractions pie chart of all overlapped regions, we found that *cis*-regulatory regions from cardiovascular disease samples were extracted, such as cardiac hypertrophy and myocardial infarction (Figure 3C, right). Overlapped regions of embryonic heart validated the marker effects of Nppa/Nppb in heart development. Additionally, factor histogram of overlapped regions validated the high chromatin activity and strong ability of super-enhancers in recruiting transcription regulators.

Identification of upstream regulators for heart failure genes

Previous studies have demonstrated that dysregulation of upstream transcription regulators of disease genes is the major cause of cardiovascular diseases. For instance, fetal genes in cardiac development have been demonstrated to regulate heart failure via mediating key biological processes, such as calcium handling, oxidative phosphorylation, switch from fatty acid to glucose metabolism, and mitochondrial dysfunction. All these fetal genes are driven in part by TFs, such as the *MEF* family, *GATA4, NTAT*, *SRF*, and *NKX2.5.*[30,31] *Cis*-Cardio integrated comprehensive transcription regulator/*cis*-regulatory element/gene pairs, and we then collected the top 20 heart failure disease genes from DisGeNET with ranked gene-disease association score (Figure S1A). As a result, *Cis*-Cardio could locate the upstream candidate *cis*-regulatory elements and TFs of these disease genes (Figure S1B). For instance, *HIF1A* is the potential target gene of heart left ventricle *H3K4me3* ChIP-seq peak "Cardio_01_1032818447," which is regulated by 53 TFs, including the known heart failure regulators SP1 and *YY1*.[32,33]

In addition, we also listed the regulatory details of the *HIF1A*-related *cis*-regulatory region (Figure S1C). Annotation results showed that this region had the enormous potential to regulate gene expression as super-enhancers and enhancers in multiple cells. Target genes of this region were also upregulated in cardiovascular diseases, especially in coronary artery disease, which is one of major causes of heart failure.

Interpretation of variations of coronary heart diseases

Previous studies have demonstrated that non-coding genetics variations could modify TF binding affinities on *cis*-regulatory elements

Figure 3. Case study-related analysis results of *Cis*-Cardio

(A) Left: input genes of transcription regulator enrichment analysis, which were cardiac marker genes collected from CellMarker. Right: bar plot of enrichment scores. (B) Results of transcription regulator enrichment analysis. (C) Left: input region of genomic region intersection analysis, which was a super-enhancer region of heart failure genes *Nppa* and *Nppb*. Right: pie plot of samples of overlapped regions. (D) Results of genomic region intersection analysis.

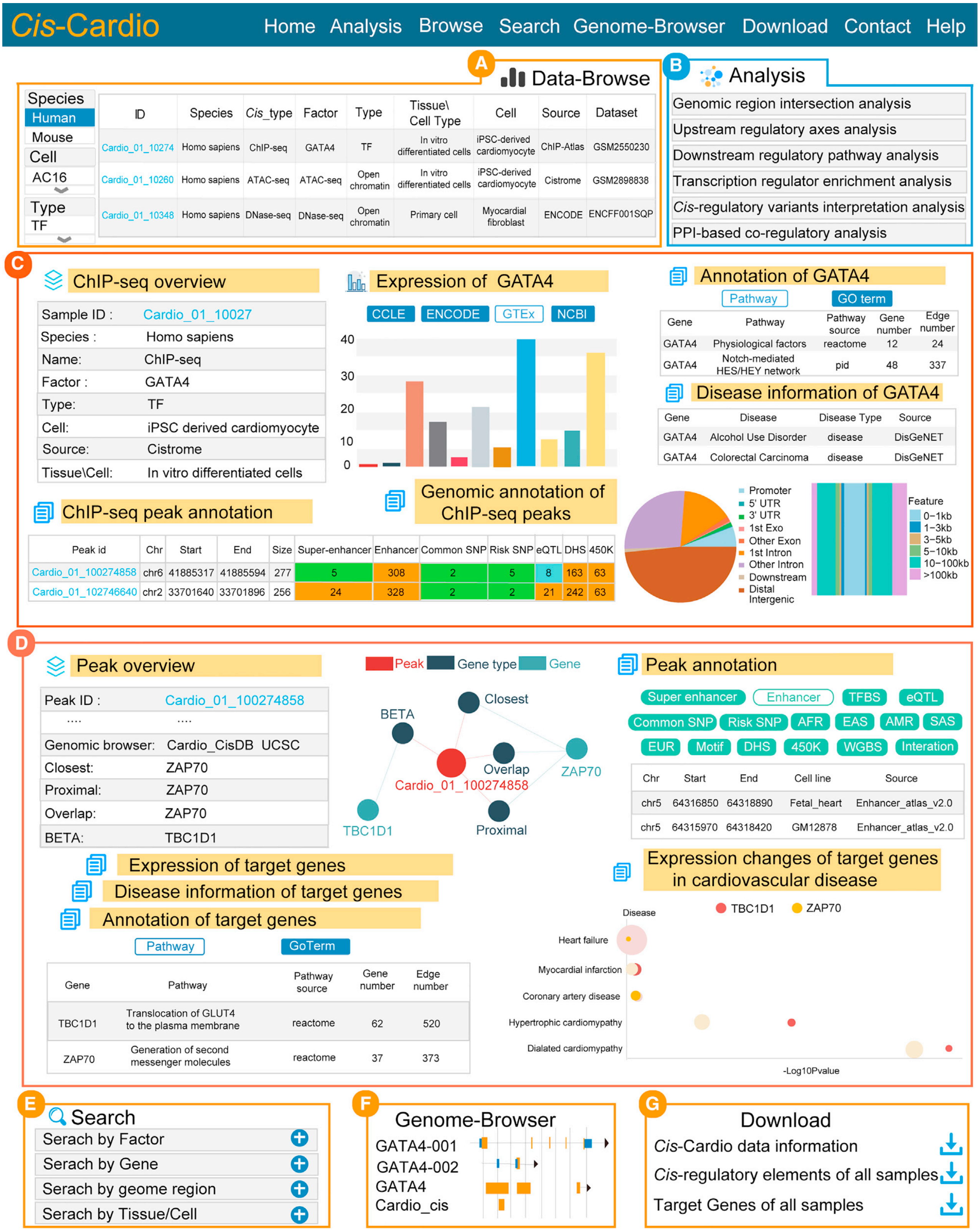

(legend on next page)

and disturb distal-proximal element connections to participate in the processes of cardiovascular diseases.[34] *Cis*-Cardio collected a large set of variants and integrated abundant annotations to interpret the potential functions of these variants. *SMAD3* is the crucial regulator of coronary heart disease. We mapped the *SMAD3*-related top variant "rs17293632" of coronary heart disease genome-wide association study (GWAS) data into *Cis*-Cardio, and the results showed that this variant was enriched in multiple *cis*-regulatory elements of coronary artery smooth muscle cell, which was consistent with previous research[35] (Figures S1D and S1E). In the element "Cardio_01_1019927866" of *JUND* ChIP-seq data, some TFs located in the variant were identified, such as *EGR1* and *FOXD3*. Furthermore, the peak detail page also presented the comprehensive TF binding information and peak annotation information for users to unveil the regulatory cues between variants and *cis*-regulatory elements.

User-friendly interface for browsing, searching, and downloading *cis*-regulatory data

Cis-Cardio provides six analysis tools and a quick browse for retrieving the *cis*-regulatory data (Figures 4A and 4B). On the browse page, users can query the factors of interest via a search box or filter the samples via a checkbox. Here, we used human samples as the filter and selected a sample of cardiac-specific TF *GATA4* as an example to introduce the performance of *Cis*-Cardio. In the browse table, users can obtain the metadata of samples, such as cell type, cell name, data source, and dataset accession number. Users can click the data ID links to the data detail page. On the detail page, users can view the comprehensive annotations of *GATA4*, including *GATA4* ChIP-seq overview, *GATA4* ChIP-seq peaks, peak annotations, and genomic peak annotation information, which are useful to help investigate the regulatory patterns of factors. For instance, the results showed that *GATA4* preferred to bind distal regions, which is coincident with previous studies.[13] For the samples of transcription regulators, *Cis*-Cardio also provided the basic annotations for transcription regulators; here, we displayed the basic annotations for *GATA4*, including *GATA4*-related gene ontology/pathway annotation, expression, and disease information (Figure 4C).

Importantly, users can click "Peak id" to obtain the details about each *cis*-regulatory region of interest. In the peak detail page, *Cis*-Cardio provided abundant detailed annotations for the *cis*-regulatory region of interest, including super-enhancer, enhancer, TF binding site, eQTL, common SNP, risk SNP, LD SNPs, DNase I hypersensitive sites, binding TFs predicted by motif, methylation sites of 450k array, methylation sites of whole-genome bisulfite sequencing, and 3D chromatin interactions. All the annotation information could help users to understand the primary causes of diseases and identify the potential synergetic regulatory axis for clinical therapeutics. *Cis*-Cardio provided the predictive downstream genes of the *cis*-regulatory peak based on three methods. Target gene differential expression in cardiovascular diseases and function annotation were also embedded in the server, which could help users to screen the disease gene efficiently. Moreover, users can view the *cis*-regulatory region by genome browser and download the data of interest in the "Download" section (file descriptions are provided in the supplemental information).

DISCUSSION

Recently, more and more attention has been paid to investigate cardiovascular-specific gene transcription programs based on dissection of the communicating cues between *cis*-regulatory elements and transcription regulators.[10,36] With the development of high-throughput techniques, the volume of cardiovascular-related omics data has accumulated rapidly, especially epigenomics and transcriptome data. However, it remains a challenge to integrate and process the data from multiple perspectives. And it is necessary to develop web tools that comprised the regulation data and provided diverse analysis functions to meet the needs. Here, we developed the *Cis*-Cardio platform, which aims to document a large number of available resources of cardiovascular-related *cis*-regulatory data and to annotate and uncover the comprehensive mechanisms at the *cis*-regulation level. The current version of *Cis*-Cardio cataloged a total of 45,382,361 genomic regions from 1,013 human and mouse epigenetic datasets, including ATAC-seq, DNase-seq, Histone ChIP-seq, TF/TcoF ChIP-seq, RNA polymerase ChIP-seq, and Cohesin ChIP-seq. Moreover, to summarize the potential cardiovascular-related *cis*-regulatory regions, we merged all the *cis*-regulatory regions of each sample into an integrated region set with peak frequency using BEDTools (Figure S2). As a result, 1,395,462 unique regions were merged and provided in the "Download" page of the server. Users can find the potential hot regions of interest based on the high peak frequencies. *Cis*-Cardio is the first resource for investigating the cardiovascular-related candidate *cis*-regulatory elements, with the largest human and mouse samples and the most comprehensive annotation information. We provide a convenient platform for researchers to explore regulated information about candidate *cis*-regulatory elements and candidate *cis*-regulatory element-associated regulatory analyses.

Cis-Cardio mainly includes the remarkable features and advantages as follows: (1) *Cis*-Cardio provides comprehensive cardiovascular-specific *cis*-regulatory regions. A total of 45,382,361 genomic regions from 1,013 human and mouse epigenetic datasets were curated in the current version. (2) *Cis*-Cardio provides useful and full-featured online analysis tools: *Cis*-Cardio provides six analyses to investigate in depth the role of candidate *cis*-regulatory elements. (3) *Cis*-Cardio provides visualization and download of multiple analysis results. (4) An interface exists for conveniently retrieving *cis*-regulatory data

Figure 4. Main interfaces and usage of *Cis*-Cardio

(A) Data-Browse page of *cis*-regulatory data. (B) Six analysis tools are provided. (C) Data interface of human GATA4 in *Cis*-Cardio, including GATA4 ChIP-seq data overview, detailed interactive table of ChIP-seq information, disease information, function annotation, expression, and peak annotation. (D) Data interface of ChIP-seq peak of interest, including genomic locus, target gene assignment, target gene network, peak annotation, and target gene annotation. (E) Four search portals. (F) Genome browser interface. (G) Download interface.

on the “Search” page. *Cis*-Cardio provides four query search methods for users to obtain the *cis*-regulatory data. (5) *Cis*-Cardio provides a user-friendly “Data-Browse” page. Users may further click on the “ID” to view candidate *cis*-regulatory elements for a given sample. (6) *Cis*-Cardio embeds a personalized genome browser with intuitive data visualization. Moreover, we also validated that *Cis*-Cardio has the ability to identify upstream regulators of cardiac marker genes and to identify important genome regions based on integration of epigenetics data.

The current version of *Cis*-Cardio processed a large number of cardiovascular-specific candidate *cis*-regulatory elements. However, we also found that the elements from the transcription regulator were sparse. With the data increased, in the future updates, we will extend the scale of the candidate *cis*-regulatory elements from transcription regulator datasets and add more annotations. And we will develop a novel analysis tool to infer more transcription regulators. *Cis*-Cardio is a user-friendly platform to analyze, query, browse, and visualize information associated with *cis*-regulatory elements. We believe that *Cis*-Cardio could become a useful and effective platform for exploring potential functions and analyzing regulation of candidate *cis*-regulatory elements in cardiovascular diseases.

MATERIALS AND METHODS

Cardiovascular-related *cis*-regulation datasets

Previous studies have released abundant high-throughput data to investigate *cis*-regulation in the field of cardiovascular disease, such as Histone ChIP-seq, ATAC-seq, scATAC-seq, DNase-seq, TF ChIP-seq, TcoF ChIP-seq, Cohesin ChIP-seq, and RNA polymerase ChIP-seq. In this study, we focused on dissecting these *cis*-regulation data and manually collected 1,013 cardiovascular-related *cis*-regulation datasets with binding peaks of human and mouse from ENCODE, ChIP-Atlas scEnhancer, and Cistrome. Next, liftOver (http://genome.ucsc.edu/cgi-bin/hgLiftOver) software was used to normalize and covert all the peaks into hg19 (human) and mm10 (mouse) genome versions. We also provided an online liftOver tool to convert genome coordinates from hg19 to hg38. All *cis*-regulatory regions from each sample were merged into the unique *cis*-regulatory regions by BEDTools with default parameters. All the biosample information is provided in Table S1.

Upstream annotations of *cis*-regulatory regions

In the current version of *Cis*-Cardio, we embedded a large number of (epi-) genetic annotations in *cis*-regulatory regions, such as super-enhancer, enhancer, TFBS, methylation sites, common SNPs, risk SNPs, eQTLs, histone modifications, and 3D chromatin interactions. All the data sources are listed in Table S2.

Super-enhancer and enhancer

Cis-regulatory regions contain several types of DNA regulatory elements, including proximal promoters and distal enhancers. Notably, distal enhancers, especially super-enhancers, are considered to play prominent roles in driving cell-specific gene expression programs. Here, to annotate the enhancers and super-enhancers within cardiovascular *cis*-regulatory regions, we firstly collected the enhancer data from EnhancerAtlas,[37] HACER,[38] ENCODE,[16] FANTOM5,[39] DENDB,[40] and ENdb,[41] including 14,797,266 human enhancers and 439,092 mouse enhancers. Secondly, for super-enhancer annotations, we manually processed *H3K27ac* ChIP-seq data from ENCODE, Roadmap, NCBI GEO/SRA, and Genomics of Gene Regulation Project. In brief, we executed Bowtie software for each *H3K27ac* ChIP-seq profile and ran MACS software to call all active peaks. ROSE was used to identify super-enhancer regions. Moreover, we also collected super-enhancers of human and mouse from SEA[42] and dbSuper.[43] As a result, 2,678,273 human super-enhancers and 11,609 mouse super-enhancers were embedded in the current *Cis*-Cardio.

TF binding sites

Cis-regulatory regions have a strong ability to recruit TFs to exert regulatory functions for downstream genes. To uncover the specific TF binding events of each *cis*-regulatory region, we performed Find Individual Motif Occurrences pipeline to call the motif binding sites in these regions for ~700 TFs.[44] In detail, we curated more than 3,000 DNA binding motifs from the TRANSFAC and MEME suite, which collected from JASPAR CORE 2020 vertebrates, Homeodomains, Jolma2013, UniPROBE, and Wei2010. Significant TF binding sites within regions were defined with an optimized p value threshold of 1e–6 from motif analysis.[19] As supplementary, we collected 5,547,656 human TFBSs and 2,858,356 mouse TFBSs from UCSC and performed BEDTools to reserve the intersected TFBSs within *cis*-regulatory regions.

SNPs/linkage disequilibrium SNPs/risk SNPs/eQTLs

Variants of the *cis*-regulatory regions determine the TF binding affinities and participate in the downstream gene transcription program. To fulfill the annotations of SNPs of *cis*-regulatory regions, we downloaded 38,063,729 human common SNPs from dbSNP and used VCFTools (v0.1.13) to screen SNPs with a minimum allelic frequency (MAF) > 0.05. Plink (v1.9) was used to call the LD SNPs (r^2 = 0.8) of five super-populations (African, Ad Mixed American, East Asian, European, and South Asian). Meanwhile, we also obtained 264,514 human risk SNPs from the GWAS Catalog and GWASdb v2 and collected 2,886,133 human eQTLs from PancanQTL, seeQTL, SCAN, and Oncobase.[45–47]

Chromatin interaction/DHS/methylation

Emerging evidence has demonstrated that chromatin marks can help to uncover the regulatory effects and mechanisms between *cis*-regulatory regions, and downstream genes mark interaction data, such as DNA chromatin interaction, DNase activity, and DNA methylation states. In the current server, we downloaded the chromatin interaction data from 4DGenome and Oncobase, which included data from ChIA-PET 3C, 4C, 5C, and Hi-C. DHS annotation data of *cis*-regulatory regions were downloaded from UCSC and ENCODE. In total, 69,860,705 human DHSs of 293 samples and 9,802,229 mouse DHSs of 56 samples were obtained. In addition, we also obtained DNA methylation states of 30,392,523 methylation sites of 450k array

and 166,855,665 methylation sites of whole-genome shotgun bisulfite sequencing from ENCODE.

Functional annotations of TFs and TcoFs

To characterize the biological functions of TFs and TcoFs, we provided more annotation information, including TF/TcoF-mediated pathway, gene ontology, expression, and disease from multiple sources. In brief, we obtained TF/TcoF expression profiles from NCBI, GTEx, ENCODE, and FANTOM5. The experimentally validated TF/TcoF-disease relationships were obtained from DisGeNET, GAD, and MGI. Moreover, TF/TcoF-related pathways were downloaded from our previous study ComPAT, which curated 2,169 human and mouse pathways from 10 resources, including KEGG, Reactome, NetPath, WikiPathways, PANTHER, PID, HumanCyc, CTD, SMPDB, and INOH. The pathway gene set is provided in Table S3.

Intersection analysis between genomic regions was performed by BEDTools with the command: bedtools intersect -a query.bed -b annotation.bed -f 1E-9 -wa -wb -bed -u > result.bed.

Cis-regulatory region-related downstream target genes

To comprehensively characterize the regulatory details of *cis*-regulatory regions, we embedded multiple methods to identify the downstream target genes of each region. For all *cis*-regulatory data in bed format files, we firstly used a python script (ROSE geneMapper.py) to annotate *cis*-regulatory region-related target genes. Notably, target genes of three strategies of ROSE (overlap, proximal, and closest) were merged. Secondly, we also used binding and expression target analysis minus (BETA minus) to identify *cis*-regulatory region downstream genes.[48] Importantly, we also embedded the Activity-by-Contact model to optimize the target genes of *cis*-regulatory elements, which is a high-confidence method by integrating histone modification and chromatin contact.[49] Target genes from the above methods were integrated and used for further analysis.

Differentially expressed gene annotations of *cis*-regulatory regions

To investigate the regulatory axis of *cis*-regulatory regions in multiple cardiovascular diseases, we have collected and processed gene expression profiles and identified gene differential expression information of eight major cardiovascular diseases, including heart failure, hypertrophic/dilated cardiomyopathy, myocardial infarction, coronary artery disease, cardiac hypertrophy, and pulmonary arterial hypertension. Briefly, we obtained the original expression profiles from GEO supplementary tables. Then we divided the samples into two groups (control group and disease group) based on the sample label. Expression data of microarrays were performed with log2 transform function, and then we identified gene differential information via SAM test, which was a non-parameter test for differential gene analysis. RNA-seq data were processed by DEseq2 with raw count matrix. p values were adjusted via false discovery rate method.

Novel online analysis tools for deciphering regulatory cues of cardiovascular diseases

Dysfunction of gene-mediated downstream regulatory axes is considered the cause and potential therapeutic target of multiple cardiovascular diseases. Gene expression is determined by upstream transcription regulation programs, such as SNP, transcription regulator activity, and DNA regulatory elements states. To help biologists investigate the regulatory mechanisms of cardiovascular diseases in the aspect of transcriptional regulation, we integrated multi-omics data and developed six online analysis tools as follows.

Genomic region intersection analysis

Genomic region intersection analysis tool could identify the *cis*-regulatory regions that overlapped with the user's genomic regions of interest. Briefly, users can upload a "bed" format file of genomic regions or a region list, choose the species (human or mouse), and set overlap size (intersection size ratio between input regions and background regions) to identify potential *cis*-regulatory regions that locate at the similar genomic regions of input regions. *Cis*-Cardio will display all the *cis*-regulatory regions that overlap with the input regions, and users can also obtain detailed annotations of the overlapped regions, such as *cis*-regulation type, peak information, target gene, and cell type information.

Upstream regulatory axes analysis

Upstream regulatory axes analysis tool was developed for identification of upstream regulatory mechanisms for genes of interest, and it can list the input genes related *cis*-regulatory regions and binding TFs. Users can submit a gene list to *Cis*-Cardio to map the target genes of all candidate *cis*-regulatory elements. If the submitted genes are the target genes of a *cis*-regulatory element, *Cis*-Cardio will extract the upstream regulatory TFs of the *cis*-regulatory element and form the TFs/candidate *cis*-regulatory elements/submitted genes regulatory axes.

Downstream regulatory pathway analysis

TFs are usually located at the terminal of the signal pathways. Users can submit a gene list, and *Cis*-Cardio will identify enriched pathways in up to 10 pathway databases via a hypergeometric test. The p value of the enriched pathway was measured as follows:

$$p - value = 1 - \sum_{i=0}^{r-1} \frac{\binom{t}{i}\binom{m-t}{n-i}}{\binom{m}{n}}$$

Here, m represents the total number of genes in all pathways, t represents the number of input genes, n represents the number of genes of each pathway, and r represents the number of overlap genes between input genes and each pathway gene.

In each pathway, *Cis*-Cardio will locate the terminal TFs and extract the terminal TF-bound downstream candidate *cis*-regulatory

elements. Therefore, users can find the submitted genes/pathway/TFs/candidate *cis*-regulatory element regulatory axes using this analysis tool.

Transcription regulator enrichment analysis

Transcription regulator enrichment analysis tool provides an enrichment function to find the upstream regulators of input genes based on epigenomics data. Users can submit a gene list to *Cis*-Cardio to identify the upstream transcription regulators and epigenetics factors based on a hypergeometric test. The background TR-target gene pairs were constructed from the data based on two target gene assignment strategies. *Cis*-Cardio will display all the datasets that overlap with the input genes via the hypergeometric test and Jaccard index. Furthermore, we also provided the genome region-based transcription regulator enrichment analysis via LOLA. Users can submit regions of interest to enrich the transcription regulators.

Cis-regulatory variants interpretation analysis

Variants that locate at the *cis*-regulatory regions determine the binding affinity of TFs and participate in the gene expression programs. *Cis*-regulatory variants interpretation analysis can quickly map cardiovascular-related candidate *cis*-regulatory elements and TF binding sites that contain variants of interest. Users can submit a variant name (such as rs10817286) to *Cis*-Cardio to extract the downstream peaks that locate the variant. Previous studies revealed that variants determine the TF binding ability in DNA regulatory elements.[34] Here, users can obtain the TF binding sites in cell-specific peaks via motif analysis.

PPI-based co-regulatory analysis

It is important to decipher the regulatory axes of the regulatory proteins that are located in the cell nucleus, as they can exert functions by regulating transcription regulators. Here, users can submit a gene list (nuclear protein of interest) to find the direct TFs based on a protein-protein interaction (PPI) network. Users can also obtain the network topological importance score of the input genes and the downstream regulatory data of the interactive transcription regulators.

Server development environment

The current version of *Cis*-Cardio was developed using MySQL 5.7.17 (http://www.mysql.com), and it runs on a Linux-based Apache Web server (http://www.apache.org). PHP 7.0 (http://www.php.net) was used for server-side scripting. The interactive interface was designed and built using Bootstrap v3.3.7 (https://v3.bootcss.com) and JQuery v2.1.1 (http://jquery.com). ECharts (https://www.echartsjs.com/) and Highcharts (https://www.highcharts.com.cn/) were used as graphical visualization frameworks. The genome browser was developed based on JBrowser.[50] We recommend using a modern web browser that supports the HTML5 standard, such as Firefox, Google Chrome, Safari, Opera, or IE 9.0+, for the best display.

DATA AND CODE AVAILABILITY

All data supporting the findings of this study are available within the paper and online *Cis*-Cardio server (http://www.licpathway.net/Cis-Cardio/index.html).

SUPPLEMENTAL INFORMATION

Supplemental information can be found online at https://doi.org/10.1016/j.omtn.2023.07.030.

ACKNOWLEDGMENTS

We thank potential reviewers for their insightful suggestions and comments on this paper. This work was supported by National Natural Science Foundation of China (62171166); Research Foundation of the First Affiliated Hospital of University of South China for Advanced Talents (20210002-1005 USCAT-2021-01); China Postdoctoral Science Foundation (2019M661311); Natural Science Foundation of Hunan Province (2023JJ30547, 2023JJ40594, 2023JJ30536); Scientific Research Fund Project of Hunan Provincial Health Commission (no. 20201920); Special Funds for the Construction of Innovative Provinces in Hunan (no. 2020SK4008); and Clinical Research 4310 Program of the University of South China (no. 20214310NHYCG03, 20224310NHYCG05).

AUTHOR CONTRIBUTIONS

C.L. and H.T. developed the concepts. C.S., Y.Z., H.H., Y.W., X.Z., G.Z., M.X., and C.F. wrote the manuscript and performed data analysis. Q.W., F.Q., D.S., J.Z., and J.L. performed data analysis.

DECLARATION OF INTERESTS

The authors declare that they have no competing financial interests.

REFERENCES

1. Lee, T.I., and Young, R.A. (2013). Transcriptional regulation and its misregulation in disease. Cell *152*, 1237–1251.
2. Field, A., and Adelman, K. (2020). Evaluating Enhancer Function and Transcription. Annu. Rev. Biochem. *89*, 213–234.
3. Rickels, R., and Shilatifard, A. (2018). Enhancer Logic and Mechanics in Development and Disease. Trends Cell Biol. *28*, 608–630.
4. Signor, S.A., and Nuzhdin, S.V. (2018). The Evolution of Gene Expression in cis and trans. Trends Genet. *34*, 532–544.
5. Zabidi, M.A., and Stark, A. (2016). Regulatory Enhancer-Core-Promoter Communication via Transcription Factors and Cofactors. Trends Genet. *32*, 801–814.
6. Feng, C., Song, C., Liu, Y., Qian, F., Gao, Y., Ning, Z., Wang, Q., Jiang, Y., Li, Y., Li, M., et al. (2020). KnockTF: a comprehensive human gene expression profile database with knockdown/knockout of transcription factors. Nucleic Acids Res. *48*, D93–D100.
7. Zhang, Y., Song, C., Zhang, Y., Wang, Y., Feng, C., Chen, J., Wei, L., Pan, Q., Shang, D., Zhu, Y., et al. (2022). TcoFBase: a comprehensive database for decoding the regulatory transcription co-factors in human and mouse. Nucleic Acids Res. *50*, D391–D401.
8. Zheng, R., Wan, C., Mei, S., Qin, Q., Wu, Q., Sun, H., Chen, C.H., Brown, M., Zhang, X., Meyer, C.A., and Liu, X.S. (2019). Cistrome Data Browser: expanded datasets and new tools for gene regulatory analysis. Nucleic Acids Res. *47*, D729–D735.
9. Wang, S., Zang, C., Xiao, T., Fan, J., Mei, S., Qin, Q., Wu, Q., Li, X., Xu, K., He, H.H., et al. (2016). Modeling cis-regulation with a compendium of genome-wide histone H3K27ac profiles. Genome Res. *26*, 1417–1429.
10. Hocker, J.D., Poirion, O.B., Zhu, F., Buchanan, J., Zhang, K., Chiou, J., Wang, T.M., Zhang, Q., Hou, X., Li, Y.E., et al. (2021). Cardiac cell type-specific gene regulatory programs and disease risk association. Sci. Adv. *7*, eabf1444.

11. Huang, S., Li, X., Zheng, H., Si, X., Li, B., Wei, G., Li, C., Chen, Y., Chen, Y., Liao, W., et al. (2019). Loss of Super-Enhancer-Regulated circRNA Nfix Induces Cardiac Regeneration After Myocardial Infarction in Adult Mice. Circulation *139*, 2857–2876.

12. Galang, G., Mandla, R., Ruan, H., Jung, C., Sinha, T., Stone, N.R., Wu, R.S., Mannion, B.J., Allu, P.K.R., Chang, K., et al. (2020). ATAC-Seq Reveals an Isl1 Enhancer That Regulates Sinoatrial Node Development and Function. Circ. Res. *127*, 1502–1518.

13. Ang, Y.S., Rivas, R.N., Ribeiro, A.J.S., Srivas, R., Rivera, J., Stone, N.R., Pratt, K., Mohamed, T.M.A., Fu, J.D., Spencer, C.I., et al. (2016). Disease Model of GATA4 Mutation Reveals Transcription Factor Cooperativity in Human Cardiogenesis. Cell *167*, 1734–1749.e22.

14. Smemo, S., Campos, L.C., Moskowitz, I.P., Krieger, J.E., Pereira, A.C., and Nobrega, M.A. (2012). Regulatory variation in a TBX5 enhancer leads to isolated congenital heart disease. Hum. Mol. Genet. *21*, 3255–3263.

15. Wang, F., Bai, X., Wang, Y., Jiang, Y., Ai, B., Zhang, Y., Liu, Y., Xu, M., Wang, Q., Han, X., et al. (2021). ATACdb: a comprehensive human chromatin accessibility database. Nucleic Acids Res. *49*, D55–D64.

16. Luo, Y., Hitz, B.C., Gabdank, I., Hilton, J.A., Kagda, M.S., Lam, B., Myers, Z., Sud, P., Jou, J., Lin, K., et al. (2020). New developments on the Encyclopedia of DNA Elements (ENCODE) data portal. Nucleic Acids Res. *48*, D882–D889.

17. Hammal, F., de Langen, P., Bergon, A., Lopez, F., and Ballester, B. (2022). ReMap 2022: a database of Human, Mouse, Drosophila and Arabidopsis regulatory regions from an integrative analysis of DNA-binding sequencing experiments. Nucleic Acids Res. *50*, D316–D325.

18. Zou, Z., Ohta, T., Miura, F., and Oki, S. (2022). ChIP-Atlas 2021 update: a data-mining suite for exploring epigenomic landscapes by fully integrating ChIP-seq, ATAC-seq and Bisulfite-seq data. Nucleic Acids Res. *50*, W175–W182.

19. Wang, Y., Song, C., Zhao, J., Zhang, Y., Zhao, X., Feng, C., Zhang, G., Zhu, J., Wang, F., Qian, F., et al. (2023). SEdb 2.0: a comprehensive super-enhancer database of human and mouse. Nucleic Acids Res. *51*, D280–D290.

20. McLean, C.Y., Bristor, D., Hiller, M., Clarke, S.L., Schaar, B.T., Lowe, C.B., Wenger, A.M., and Bejerano, G. (2010). GREAT improves functional interpretation of cis-regulatory regions. Nat. Biotechnol. *28*, 495–501.

21. Gao, T., Zheng, Z., Pan, Y., Zhu, C., Wei, F., Yuan, J., Sun, R., Fang, S., Wang, N., Zhou, Y., and Qian, J. (2022). scEnhancer: a single-cell enhancer resource with annotation across hundreds of tissue/cell types in three species. Nucleic Acids Res. *50*, D371–D379.

22. Liu, B., Liu, X., Han, L., Chen, X., Wu, X., Wu, J., Yan, D., Wang, Y., Liu, S., Shan, L., et al. (2022). BRD4-directed super-enhancer organization of transcription repression programs links to chemotherapeutic efficacy in breast cancer. Proc. Natl. Acad. Sci. USA *119*. e2109133119.

23. Bal, E., Kumar, R., Hadigol, M., Holmes, A.B., Hilton, L.K., Loh, J.W., Dreval, K., Wong, J.C.H., Vlasevska, S., Corinaldesi, C., et al. (2022). Super-enhancer hypermutation alters oncogene expression in B cell lymphoma. Nature *607*, 808–815.

24. Feng, C., Song, C., Jiang, Y., Zhao, J., Zhang, J., Wang, Y., Yin, M., Zhu, J., Ai, B., Wang, Q., et al. (2023). Landscape and significance of human super enhancer-driven core transcription regulatory circuitry. Mol. Ther. Nucleic Acids *32*, 385–401.

25. Kelly, M.R., Wisniewska, K., Regner, M.J., Lewis, M.W., Perreault, A.A., Davis, E.S., Phanstiel, D.H., Parker, J.S., and Franco, H.L. (2022). A multi-omic dissection of super-enhancer driven oncogenic gene expression programs in ovarian cancer. Nat. Commun. *13*, 4247.

26. Man, J.C.K., Bosada, F.M., Scholman, K.T., Offerhaus, J.A., Walsh, R., van Duijvenboden, K., van Eif, V.W.W., Bezzina, C.R., Verkerk, A.O., Boukens, B.J., et al. (2021). Variant Intronic Enhancer Controls SCN10A-short Expression and Heart Conduction. Circulation *144*, 229–242.

27. Zhang, X., Lan, Y., Xu, J., Quan, F., Zhao, E., Deng, C., Luo, T., Xu, L., Liao, G., Yan, M., et al. (2019). CellMarker: a manually curated resource of cell markers in human and mouse. Nucleic Acids Res. *47*, D721–D728.

28. Sabari, B.R., Dall'Agnese, A., Boija, A., Klein, I.A., Coffey, E.L., Shrinivas, K., Abraham, B.J., Hannett, N.M., Zamudio, A.V., Manteiga, J.C., et al. (2018). Coactivator condensation at super-enhancers links phase separation and gene control. Science *361*, eaar3958.

29. Man, J.C.K., van Duijvenboden, K., Krijger, P.H.L., Hooijkaas, I.B., van der Made, I., de Gier-de Vries, C., Wakker, V., Creemers, E.E., de Laat, W., Boukens, B.J., and Christoffels, V.M. (2021). Genetic Dissection of a Super Enhancer Controlling the Nppa-Nppb Cluster in the Heart. Circ. Res. *128*, 115–129.

30. van Rooij, E., Sutherland, L.B., Qi, X., Richardson, J.A., Hill, J., and Olson, E.N. (2007). Control of stress-dependent cardiac growth and gene expression by a microRNA. Science *316*, 575–579.

31. Backs, J., and Olson, E.N. (2006). Control of cardiac growth by histone acetylation/deacetylation. Circ. Res. *98*, 15–24.

32. Lax, A., Soler, F., Fernandez Del Palacio, M.J., Pascual-Oliver, S., Ballester, M.R., Fuster, J.J., Pascual-Figal, D., and Asensio-Lopez, M.D.C. (2023). Silencing of microRNA-106b-5p prevents doxorubicin-mediated cardiotoxicity through modulation of the PR55alpha/YY1/sST2 signaling axis. Mol. Ther. Nucleic Acids *32*, 704–720.

33. Zhan, J., Jin, K., Ding, N., Zhou, Y., Hu, G., Yuan, S., Xie, R., Wen, Z., Chen, C., Li, H., and Wang, D.W. (2023). Positive feedback loop of miR-320 and CD36 regulates the hyperglycemic memory-induced diabetic diastolic cardiac dysfunction. Mol. Ther. Nucleic Acids *31*, 122–138.

34. Richter, F., Morton, S.U., Kim, S.W., Kitaygorodsky, A., Wasson, L.K., Chen, K.M., Zhou, J., Qi, H., Patel, N., DePalma, S.R., et al. (2020). Genomic analyses implicate noncoding *de novo* variants in congenital heart disease. Nat. Genet. *52*, 769–777.

35. Turner, A.W., Hu, S.S., Mosquera, J.V., Ma, W.F., Hodonsky, C.J., Wong, D., Auguste, G., Song, Y., Sol-Church, K., Farber, E., et al. (2022). Single-nucleus chromatin accessibility profiling highlights regulatory mechanisms of coronary artery disease risk. Nat. Genet. *54*, 804–816.

36. Nim, H.T., Dang, L., Thiyagarajah, H., Bakopoulos, D., See, M., Charitakis, N., Sibbritt, T., Eichenlaub, M.P., Archer, S.K., Fossat, N., et al. (2021). A cis-regulatory-directed pipeline for the identification of genes involved in cardiac development and disease. Genome Biol. *22*, 335.

37. Gao, T., and Qian, J. (2020). EnhancerAtlas 2.0: an updated resource with enhancer annotation in 586 tissue/cell types across nine species. Nucleic Acids Res. *48*, D58–D64.

38. Wang, J., Dai, X., Berry, L.D., Cogan, J.D., Liu, Q., and Shyr, Y. (2019). HACER: an atlas of human active enhancers to interpret regulatory variants. Nucleic Acids Res. *47*, D106–D112.

39. FANTOM Consortium and the RIKEN PMI and CLST DGT, Forrest, A.R.R., Kawaji, H., Rehli, M., Baillie, J.K., de Hoon, M.J.L., Haberle, V., Lassmann, T., Kulakovskiy, I.V., Lizio, M., et al. (2014). A promoter-level mammalian expression atlas. Nature *507*, 462–470.

40. Ashoor, H., Kleftogiannis, D., Radovanovic, A., and Bajic, V.B. (2015). DENdb: Database of Integrated Human Enhancers. Database *2015*, bav085.

41. Bai, X., Shi, S., Ai, B., Jiang, Y., Liu, Y., Han, X., Xu, M., Pan, Q., Wang, F., Wang, Q., et al. (2020). ENdb: a manually curated database of experimentally supported enhancers for human and mouse. Nucleic Acids Res. *48*, D51–D57.

42. Chen, C., Zhou, D., Gu, Y., Wang, C., Zhang, M., Lin, X., Xing, J., Wang, H., and Zhang, Y. (2020). SEA version 3.0: a comprehensive extension and update of the Super-Enhancer archive. Nucleic Acids Res. *48*, D198–D203.

43. Khan, A., and Zhang, X. (2016). dbSUPER: a database of super-enhancers in mouse and human genome. Nucleic Acids Res. *44*, D164–D171.

44. Grant, C.E., Bailey, T.L., and Noble, W.S. (2011). FIMO: scanning for occurrences of a given motif. Bioinformatics *27*, 1017–1018.

45. Li, M.J., Liu, Z., Wang, P., Wong, M.P., Nelson, M.R., Kocher, J.P.A., Yeager, M., Sham, P.C., Chanock, S.J., Xia, Z., and Wang, J. (2016). GWASdb v2: an update database for human genetic variants identified by genome-wide association studies. Nucleic Acids Res. *44*, D869–D876.

46. Li, X., Shi, L., Wang, Y., Zhong, J., Zhao, X., Teng, H., Shi, X., Yang, H., Ruan, S., Li, M., et al. (2019). OncoBase: a platform for decoding regulatory somatic mutations in human cancers. Nucleic Acids Res. *47*, D1044–D1055.

47. Gong, J., Mei, S., Liu, C., Xiang, Y., Ye, Y., Zhang, Z., Feng, J., Liu, R., Diao, L., Guo, A.Y., et al. (2018). PancanQTL: systematic identification of cis-eQTLs and trans-eQTLs in 33 cancer types. Nucleic Acids Res. *46*, D971–D976.

48. Wang, S., Sun, H., Ma, J., Zang, C., Wang, C., Wang, J., Tang, Q., Meyer, C.A., Zhang, Y., and Liu, X.S. (2013). Target analysis by integration of transcriptome and ChIP-seq data with BETA. Nat. Protoc. *8*, 2502–2515.

49. Fulco, C.P., Nasser, J., Jones, T.R., Munson, G., Bergman, D.T., Subramanian, V., Grossman, S.R., Anyoha, R., Doughty, B.R., Patwardhan, T.A., et al. (2019). Activity-by-contact model of enhancer-promoter regulation from thousands of CRISPR perturbations. Nat. Genet. *51*, 1664–1669.

50. Diesh, C., Stevens, G.J., Xie, P., De Jesus Martinez, T., Hershberg, E.A., Leung, A., Guo, E., Dider, S., Zhang, J., Bridge, C., et al. (2023). JBrowse 2: a modular genome browser with views of synteny and structural variation. Genome Biol. *24*, 74.

OMTN, Volume *33*

Supplemental information

Cis-Cardio: A comprehensive analysis platform for cardiovascular-relavant *cis*-regulation in human and mouse

Chao Song, Yuexin Zhang, Hong Huang, Yuezhu Wang, Xilong Zhao, Guorui Zhang, Mingxue Yin, Chenchen Feng, Qiuyu Wang, Fengcui Qian, Desi Shang, Jian Zhang, Jiaqi Liu, Chunquan Li, and Huifang Tang

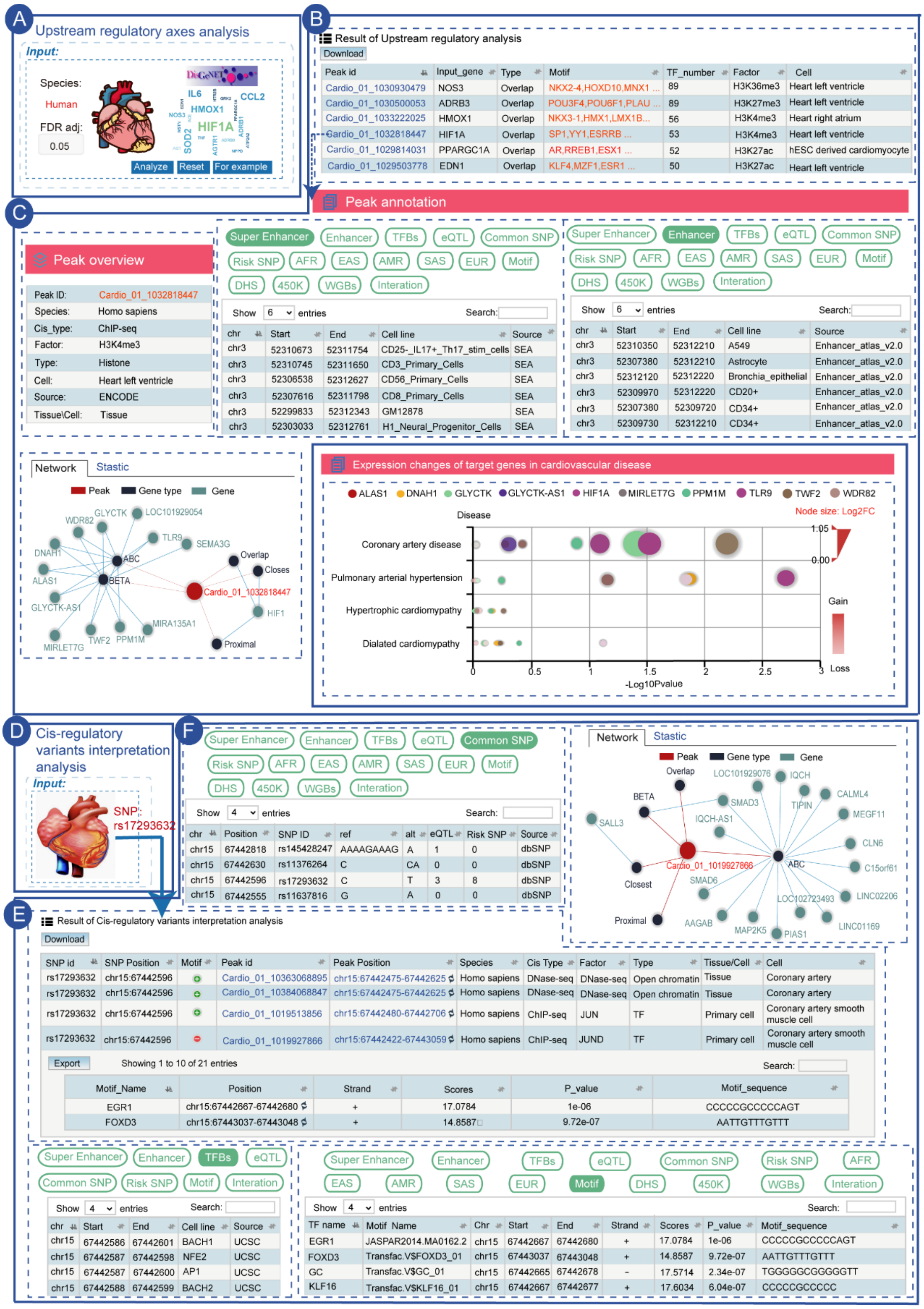

Figure S1: Additional case study related analysis results of Cis-Cardio. (A) Input genes of top 20 heart failure disease genes, which were downloaded from DisGeNET. (B) Results of Upstream regulatory axes analysis. (C) Data interface of ChIP-seq peak

of interest, including genomic locus, target gene assignment, target gene network, peak annotation and target gene annotation and differential expression information. (D) Input variant of coronary heart disease. (E) Results of Cis-regulatory variants interpretation analysis. (F) Data interface of ChIP-seq peak of interest, including TF binding sites, target gene assignment, target gene network and peak annotation.

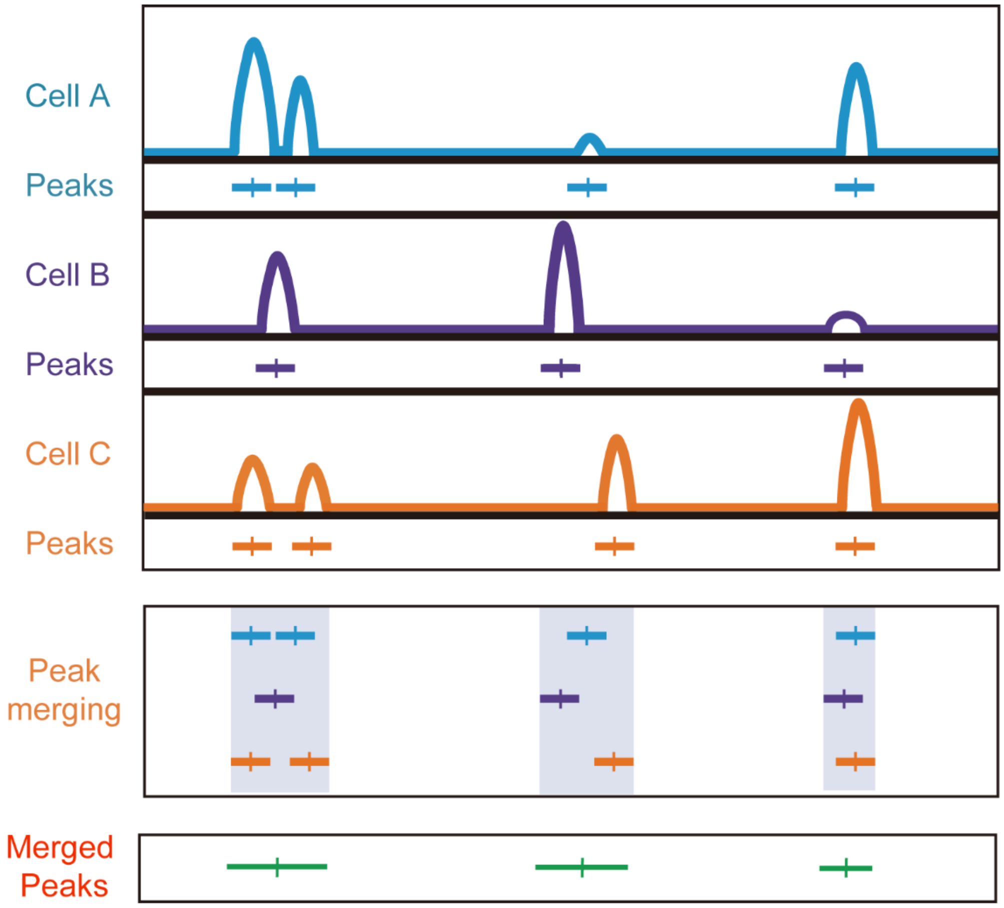

Figure S2: The protocol to merge peaks into an integrated region set based on Bedtools.

Table S1: Biosample metadata of Cis-Cardio. Sheet 1 is the metadata of samples with GEO accession number. Sheet 2 is the metadata of samples with ENCODE accession number.

Table S2: Metadata and processing protocol of (epi)genetics annotation data.

Type	Source name	Link	Version
Super Enhancer	SEdb	http://www.licpathway.net/sedb	V1
Super enhancer	SEA	http://sea.edbc.org	V3
Super enhancer	dbSuper	http://bioinfo.au.tsinghua.edu.cn/dbsuper/	V1
Enhancer	EnhancerAtlas	http://www.enhanceratlas.org/indexv2.php	V2
Enhancer	HACER	http://bioinfo.vanderbilt.edu/AE/HACER/	V1
Enhancer	FANTOM5	https://fantom.gsc.riken.jp/5/datafiles/latest/extra/Enhancers/	V1
Enhancer	DENDB	http://www.cbrc.kaust.edu.sa/dendb/	V1
Enhancer	ENdb	http://www.licpathway.net/ENdb	V1
Enhancer	ENCODE	https://www.encodeproject.org/search/?type=Experiment&status=released&award.rfa=ENCODE2	V2
Common SNP	dbSNP	http://www.ncbi.nlm.nih.gov/SNP/	dbSNP 150
risk SNP	GWASdb v2	http://jjwanglab.org/gwasdb	V2
eQTL	PancanQTL	http://bioinfo.life.hust.edu.cn/PancanQTL	V1
eQTL	seeQTL	http://www.bios.unc.edu/research/genomic_software/seeQTL/	V1
eQTL	SCAN	http://www.scandb.org	V1
eQTL	Oncobase	http://www.oncobase.biols.ac.cn	V1
Chromatin interaction	4DGenome	http://4dgenome.int-med.uiowa.edu/	V1
Chromatin interaction	Oncobase	http://www.oncobase.biols.ac.cn	V1
DHS	UCSC	https://www.genome.ucsc.edu/index.html	V1
DHS	ENCODE	https://www.encodeproject.org/search/?type=Experiment&status=released&award.rfa=ENCODE3	V3
DNA methylation	ENCODE	https://www.encodeproject.org/search/?type=Experiment&status=released&award.rfa=ENCODE3	V3
TFBS	UCSC	https://www.genome.ucsc.edu/index.html	V1

PPI	STRING	https://cn.string-db.org/	V11.5

Table S3: Pathway gene set of Cis-Cardio analysis panel.

Description of "bed format" download files

1) **Human bed file column name:** Chrome, Start, End, Peak_id,, Peak size, Number of super enhancer, Number of enhancer, Number of common SNP, Number of Risk SNP, Number of eQTL, Number of DHS, Number of 450k sites and Number of TFBS.
2) **Mouse bed file column name:** Chrome, Start, End, Peak_id,, Peak size, Number of super enhancer, Number of TFBS. Number of enhancer and Number of DHS.
